# Supplementary material for: Coordination of Di-Acetylated Histone Ligands by the ATAD2 Bromodomain
Source: Int J Mol Sci. 2021 Aug 24;22(17):9128. doi: 10.3390/ijms22179128 (PMC8430952; doi:10.3390/ijms22179128)
Supplement: Supplementary file 1 [file ijms-22-09128-s001.zip › ijms-1334383-supplementary.pdf]

Table S1: Dissociation constants and N-values of the interactions of the ATAD2 mutant bromodomains with a selection of histone tail peptides as measured by isothermal titration calorimetry.

ATAD2 Bromodomain Mutant	Ligand	K_D (μM)	N-value
Wild Type	H4 unmod	No binding	-
	H4K5ac (1–15)	39.4 ± 6.0	1.040
	H4K12ac (4–17)	95.1 ± 13.1	1.170
	H4K5acK8ac (1–10)	33.2 ± 5.9	0.938
	H4K5acK12ac (1–15)	28.4 ± 1.3	0.910
N1064A	H4 unmod	No binding	-
	H4K5ac (1–15)	No binding	-
	H4K12ac (4–17)	No binding	-
	H4K5acK8ac (1–10)	No binding	-
	H4K5acK12ac (1–15)	No binding	-
I1074A	H4 unmod	No binding	-
	H4K5ac (1–15)	No binding	-
	H4K12ac (4–17)	No binding	-
	H4K5acK8ac (1–10)	No binding	-
	H4K5acK12ac (1–15)	No binding	-
I1074Y	H4 unmod	No binding	-
	H4K5ac (1–15)	No binding	-
	H4K12ac (4–17)	No binding	-
	H4K5acK8ac (1–10)	No binding	-
	H4K5acK12ac (1–15)	No binding	-

Table S2: Secondary structure of the ATAD2 bromodomain wild-type and mutant proteins as measured by circular dichroism and calculated using the K2D3 program[1].

ATAD2 bromodomain	% α-helix	% β-strand
Wild Type	92.56%	0.47%
N1064A	92.56%	0.49%
I1074A	92.56%	0.49%
I1074Y	91.82%	0.50%

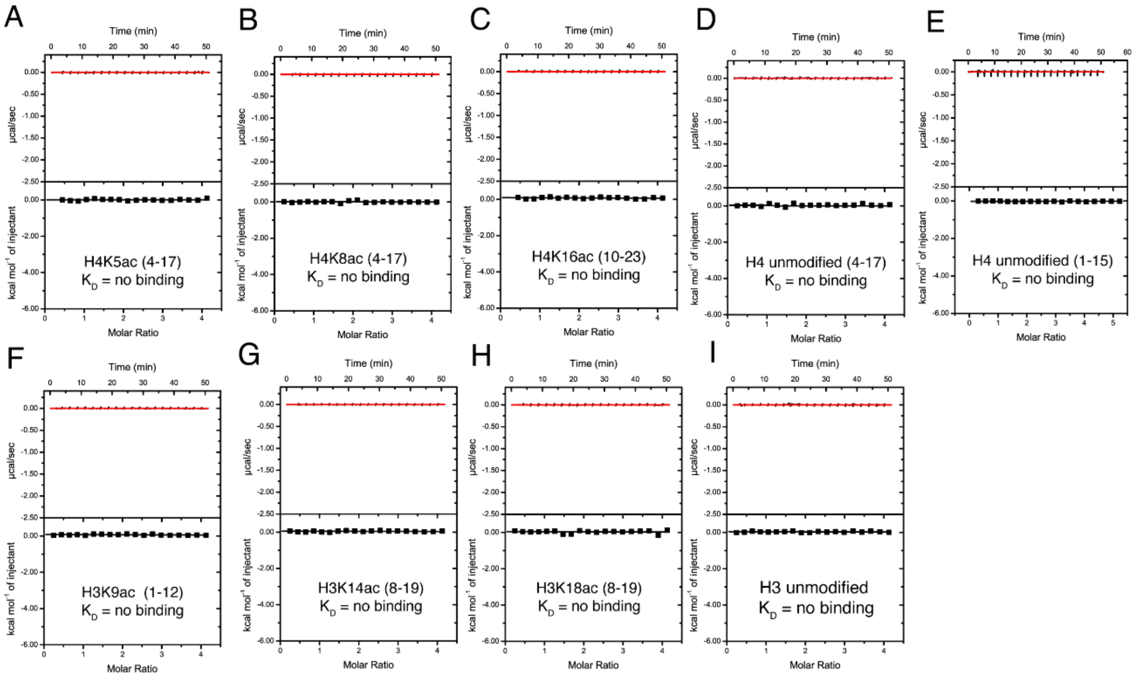

Figure S1: Isothermal titration calorimetry (ITC) assays of the ATAD2 bromodomain protein with non-binding histone ligands are shown in (**A–I**). The thermogram and integration of each peptide is shown in the top and bottom *panels*, respectively.

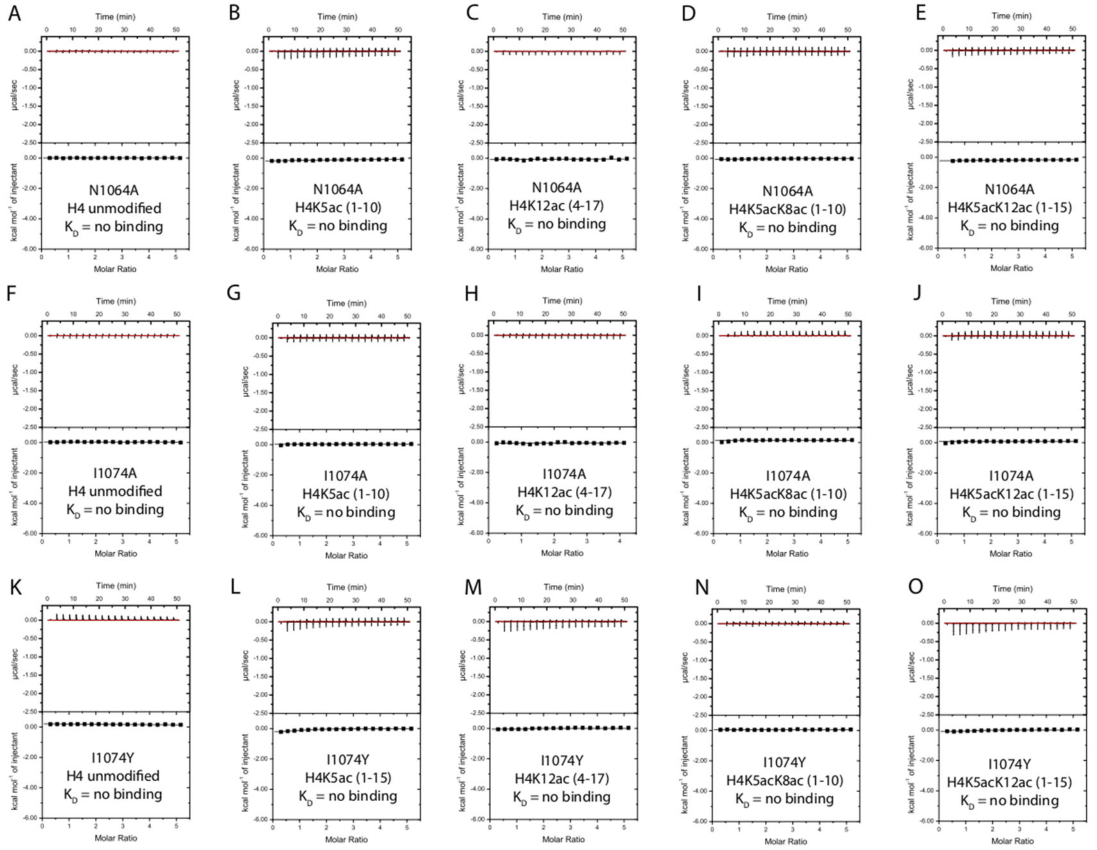

Figure S2: Isothermal titration calorimetry enthalpy plots (**A-O**) for the ATAD2 bromodomain mutant proteins upon titration of histone H4 unmodified (4–17), H4K5ac (1–15), H4K12ac (4–17), H4K5acK8ac (1–10), and H4K5acK12ac (1–15) ligands.

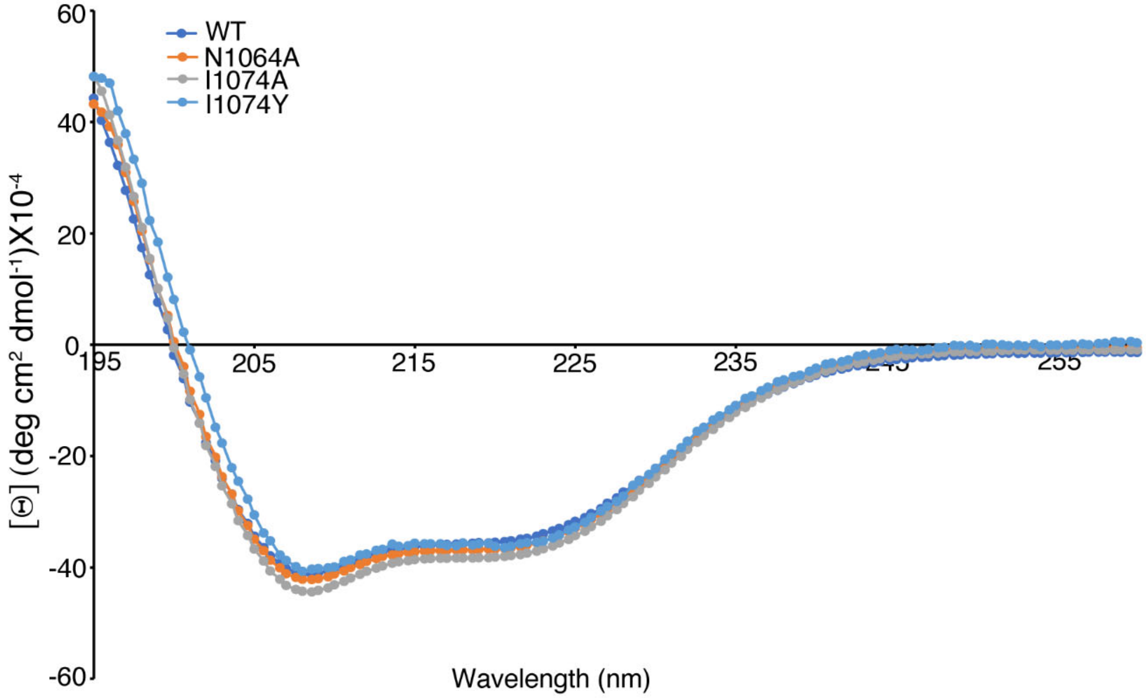

Figure S3: Circular dichroism spectra of the ATAD2 wild-type and mutant bromodomain proteins. Structural properties of the proteins are depicted in units of molar ellipticity over a range of wavelengths.

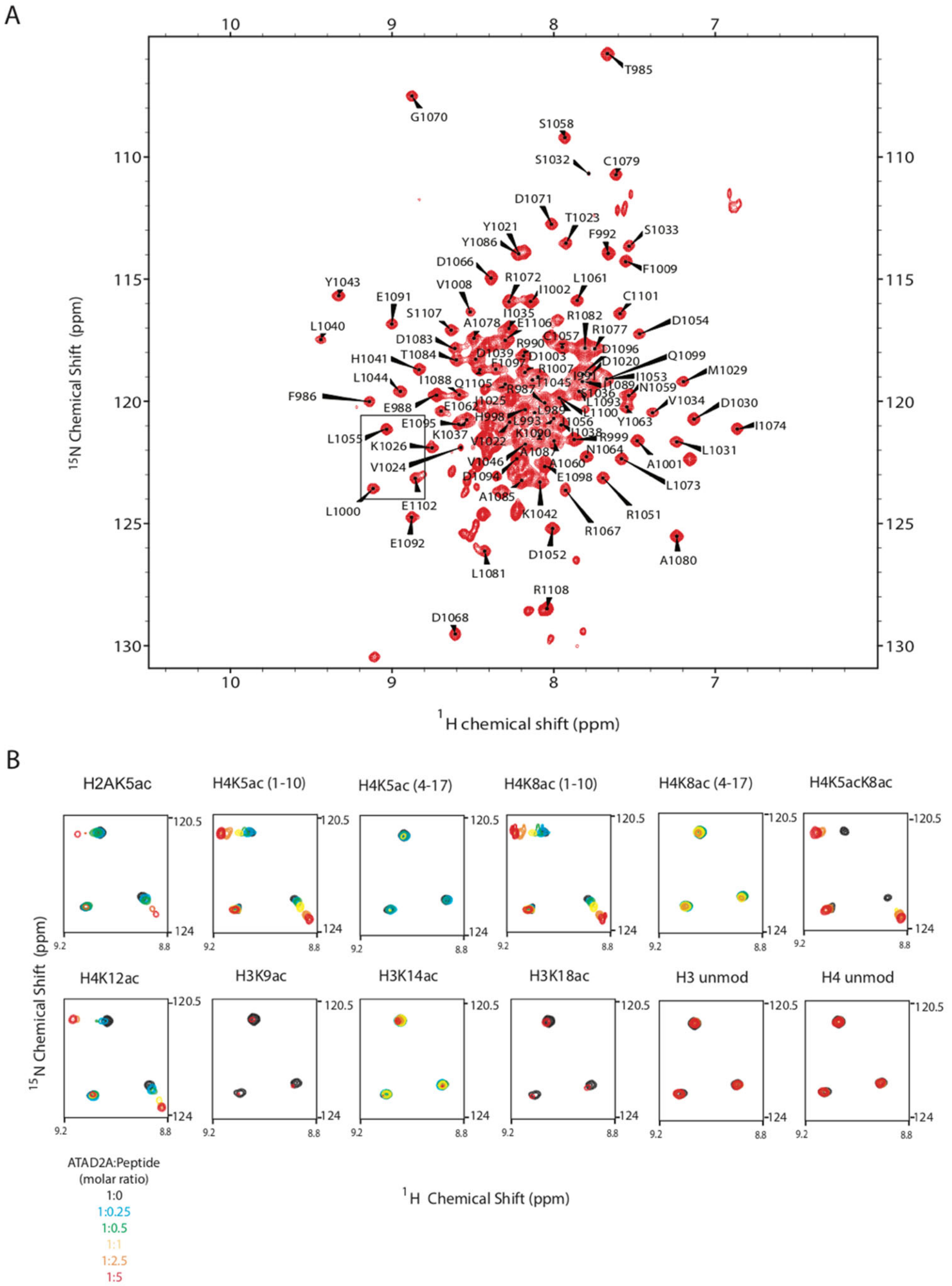

Figure S4: Nuclear magnetic resonance assignment and HSQC titration data for the ATAD2 bromodomain. (**A**) 2D ^{1}H-^{15}N HSQC spectrum of the ^{15}N-labelled ATAD2 bromodomain (BRD) showing backbone-amide assignments (**B**) The triad of residues shown in the inset A contain superimposed ^{1}H–^{15}N HSQC spectra of the ATAD2 BRD acquired with increasing concentrations of the acetylated histone tail peptides, as indicated. The spectra are color-coded according to the ratio of protein to peptide as given in the lower left corner.

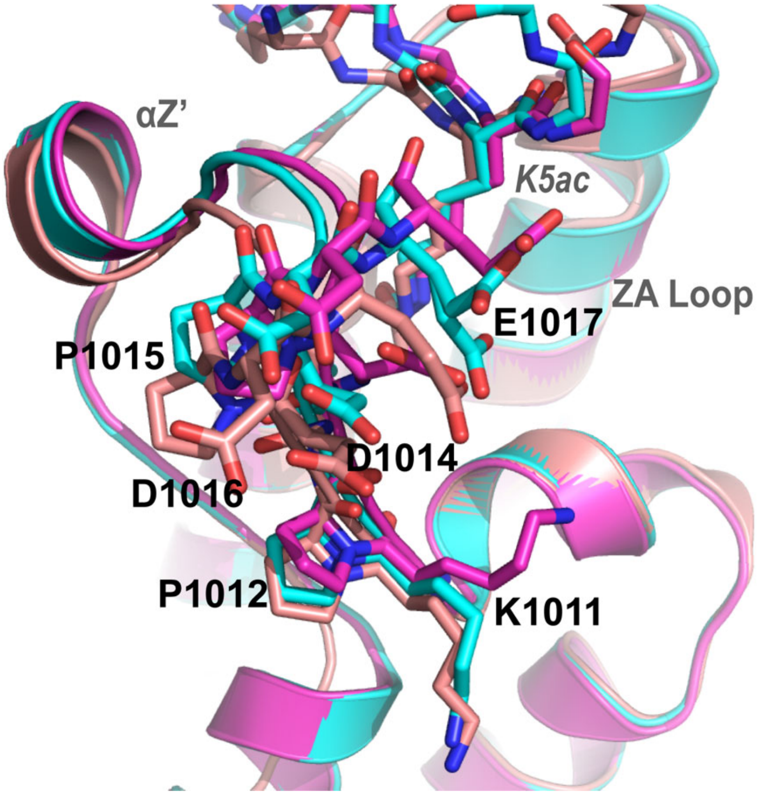

Figure S5: Structural alignment of the ATAD2 bromodomains (BRD). The ATAD2 BRD structures in complex with histone ligands H4K5acK8ac (7M98, cyan), and H4K5ac (4TT2, magenta)/(4QUU, salmon), were aligned using PyMol[2]. The ZA loop residues are displayed as sticks and labeled with residue numbers 1011–1017.

References

1. Louis-Jeune, C., Andrade-Navarro, M. A. & Perez-Iratxeta, C. (2012). Prediction of protein secondary structure from circular dichroism using theoretically derived spectra. *Proteins* **80**, 374-381.
2. DeLano, W. L. (2002). The PyMOL Molecular Graphics System. DeLano Scientific, Palo Alto, CA.
